# The attitudes of postgraduate medical students towards the curriculum by degree type: a large-scale questionnaire survey

**DOI:** 10.1186/s12909-023-04846-5

**Published:** 2023-11-16

**Authors:** Xue Jia, Yuyi Zhu, Xuelian Zhong, Qiao Wen, Deren Wang, Mangmang Xu

**Affiliations:** 1 Section of Faculty Affairs, Human Resources Department, West China School of Medicine/West China Hospital, Sichuan University, Chengdu, Sichuan Province China; 2 Department of Neurology, West China Hospital, Sichuan University, Chengdu, Sichuan Province China; 3 Center of Cerebrovascular Diseases, West China Hospital, Sichuan University, No. 37 Guo Xue Xiang, Chengdu, Sichuan Province 610041 China; 4 West China School of Nursing, Sichuan University/West China Hospital of Sichuan University, Chengdu, Sichuan Province China; 5 Department of Otolaryngology-Head & Neck Surgery, West China Hospital, Sichuan University, Chengdu, Sichuan Province China

**Keywords:** Postgraduate medical students, Curriculum, Professional degree, Academic degree

## Abstract

**Background:**

Chinese medical schools have offered both professional and academic degrees for postgraduate students. However, there is limited information about the attitudes of professional-degree and academic-degree students. We aimed to examine the attitudes of full-time postgraduate students towards the curriculum, stratified by degree type.

**Methods:**

A 29-item online questionnaire was distributed to postgraduate students in West China School of Medicine of Sichuan University in 2020. The questionnaire was designed to collect students’ demographic characteristics, attitudes towards curricular provision and content, and classroom organization. A comparison was made between groups based on degree type (academic degree versus professional degree).

**Results:**

Overall, 645 out of 908 students at West China School of Medicine completed the questionnaire. Comparing with students pursuing academic degrees, professional-degree students were more interested if the curriculum included specialized knowledge and clinical skills, and expressed concerns over the excessive compulsory courses and inadequate optional courses (p < 0.001), particularly prominent among first-year postgraduate students. Besides, a greater proportion of professional-degree students thought the curriculum was conflict with clinical work to some extent, and they also rated taking attendance in class as less reasonable (p < 0.01). Conversely, students pursuing academic degrees expressed that the courses were inadequate in interdisciplinary curriculum and had some crossover or repetition, and they assigned a higher importance rating for the curriculum when comparing professional-degree students (all p < 0.05).

**Conclusions:**

Different attitudes toward the curriculum are observed between students pursuing professional degrees and those pursuing academic degrees. This study provides benchmark data for future postgraduate course reforms in China.

**Supplementary Information:**

The online version contains supplementary material available at 10.1186/s12909-023-04846-5.

## Introduction

China, as the most populous country in the world, has a unique medical education system [1]. Traditionally, Chinese medical schools prioritized the development of academic skills, resulting to a relatively limited clinical proficiency in meeting the growing demand for healthcare [2]. Since 1998, Chinese medical schools have offered both professional and academic degrees for postgraduate students [2].

In brief, students pursuing professional master degree are required to complete courses and undergo rotations lasting for ≥ 33 months during a three-year degree program in the wards of affiliated hospitals under the supervision of senior doctors to gain standardized residency training (SRT) (similar to residency training in USA [3]), which was nationally implemented in 2015 [4–6] to help students to be qualified for medical practitioners [2]. For students pursuing professional doctorate degree, they are required to finish an unspecified number of years standardized subspecialty training (generally 1.5 years) in hospitals, as well as finishing curricular courses and performing scientific work during a 3-year doctorate program [7, 8]. Whereas students pursuing academic master degree and academic doctorate degree in clinical medicine are exclusively dedicated to scientific research [2]. Those students are expected to finish the curriculum and prioritize the training of scientific research ability [8, 9]. However, some medical institutions may arrange supplementary activities to improve the clinical skills of academic students through outpatient work under the guidance of mentors [10] and/or participation in clinical projects involving human participants [11]. These activities are designed with an understanding that the majority of those academic degree students will return to clinical work and participate in a 3-year SRT after graduation [9, 11]. Students voluntarily choosing degree type in the student recruiting system before taking the postgraduate entrance examination.

Chinese medical institutions are mostly government-owned, with the Ministry of Education guiding curriculum framework and allowing health institutions to perform education content design and other innovative curricular reforms [1]. Over the past decade, there has been much progress in movement toward autonomous curriculum design and curriculum reforms, such as development of new curricular models or courses in Chinese medical institutions [1, 12–14]. While most of the reforms are primarily focused on the undergraduate program (the so-called 5-year program) [12, 14].

However, there is limited information about the postgraduate students’ attitudes to the overall curriculum by degree type. Furthermore, although professional degree and academic degree students have different clinical requirements, they often choose the same courses needed to apply for graduate degrees, such as clinical epidemiology and clinical biostatistics, at West China School of Medicine of Sichuan University. Hence, it is necessary to compare the students’ perceptions and behavioral intentions between professional and academic degree students to provide background information for future medical course reforms.

Therefore, our research questions were if there were any different opinions concerning (1) curricular provision, (2) curricular content, and (3) classroom organization between postgraduate students pursuing professional degree and academic degree by conducting a large-scale questionnaire survey at West China School of Medicine of Sichuan University.

## Methods

We carried out a 29-item online questionnaire survey which was distributed to postgraduate students in West China School of Medicine of Sichuan University from November 2020 to December 2020 to understand the attitudes of medical students towards the curriculum. Questions were grouped into the four categories: demographic characteristics of students, attitudes towards curriculum provision, curricular content, and classroom organization. Additionally, two open-ended questions were designed to ask participants to indicate their additional opinions on classroom organization and curriculum management. We had extensive discussions and made some refinements to the questionnaire before its formal use. The items of the questionnaire were designed by reviewing Annual Report on China Graduate Education and previous published studies [15, 16]. This study was approved the by the Ethics Committee on Biomedical Research, West China Hospital of Sichuan University.

### Study population

In our present study, we enrolled master students pursuing either a professional degree or an academic degree, as well as doctorate student pursuing either of the two degrees. In the context that there are two main types of the “5 + 3” model which is the main body of Chinese clinical medical education system, where the first one involves a 5-year undergraduate education with a bachelor’s degree followed by a 3-year of SRT, and the second one encompasses a 5-year undergraduate education and then a comprehensive 3-year professional master degree program (including a 33-month rotation to gain SRT) [4]. In our present study, master students pursuing professional degree refer to the second one.

### Subgroup analysis

Considering the significant differences in the work intensity, thinking style between non-surgical and surgical departments, it is probable that the students in the two types of departments hold different attitudes towards curriculum. To investigate this further, we performed a subgroup analysis to examine the attitudes of postgraduate students separately in non-surgical and surgical departments. In the present study, the non-surgical departments included Psychiatry and Mental Health, Rehabilitation Medicine and Physical Therapy, Geriatrics, Maternal-Neonatal Medicine, Internal Medicine, Dermatology and Venereology, General Medicine, Neurology, Clinical Discipline of Integrated Traditional Chinese and Western Medicine, and Oncology. The surgical departments included Otolaryngology, Obstetrics and Gynecology, Surgery, Ophthalmology, Sports Medicine, and Anesthesiology. The attitudes of medical students towards the curriculum were separately analyzed by degree type within each of the two subgroups.

### Questionnaire validity

To develop the questionnaire, we generated a set of items with nominal variables collect students’ demographic characteristics, as well as “reasons why the curriculum is important”, “feedback on curriculum development”, “comments on the curriculum content”, “students’ expected evaluation mode for final exam”, “favorite courses”, “unfavorite courses”, and “reasons why arrive late or leave early in class” with clear language (see Table 1). Four ordinal questions were designed to collect the students’ opinions on “What is the level of difficulty of the curriculum”, “How often do you arrive late or leave early in class”, “To what extent does using electronic devices in class affect your learning”. “What is your opinion on class attendance policy”.

**Table 1:** Students’ characteristics and attitudes towards the curriculum by degree type

Variable	Overall (n = 645)	Academic degree (n = 328)	Professional degree (n = 317)	P value
**Sex**, male, n (%)	203 (31.5)	99 (30.2)	104 (32.8)	0.473
Grade Level*				
2020, n (%)	534 (82.9)	276 (84.4)	258 (81.4)	0.295
2019, n (%)	69 (10.7)	35 (10.7)	34 (10.7)	
2018, n (%)	41 (6.4)	16 (4.9)	25 (7.9)	
**Postgraduate degree**, doctorate degree, n (%)	160 (24.8)	78 (23.8)	82 (25.9)	0.540
**Reasons why the curriculum is important**, n (%)				
Minimum credit requirements for applying for graduate degrees	354 (54.9)	185 (56.4)	169 (53.3)	0.430
Acquiring knowledge and skills for scientific research	540 (83.7)	276 (84.1)	264 (83.3)	0.766
Enhancing English proficiency for international academic exchange and academic paper writing	356 (55.2)	172 (52.4)	184 (58.0)	0.152
Acquiring specialized knowledge and clinical skills	277 (42.9)	122 (37.2)	155 (48.9)	**0.003**
Promoting multidisciplinary and interdisciplinary education and cultivating top talent	154 (23.9)	99 (30.2)	55 (17.4)	**< 0.001**
Developing knowledge of the humanities and social sciences	80 (12.4)	46 (14.0)	34 (10.7)	0.204
**Feedback on curriculum development**, n (%)				
Excessive compulsory courses and inadequate optional courses	165 (25.6)	55 (16.8)	110 (34.7)	**< 0.001**
Inadequate curriculum for developing scientific research abilities	335 (51.9)	167 (50.9)	168 (53.0)	0.597
Inadequate curriculum for acquiring clinical skills	228 (35.3)	106 (32.3)	122 (38.5)	0.101
Inadequate humanistic curriculum	125 (19.4)	73 (22.3)	52 (16.4)	0.060
Inadequate interdisciplinary curriculum	170 (26.4)	99 (30.2)	71 (22.4)	**0.025**
The course content has some overlap or repetition to some extent	171 (26.5)	108 (32.9)	63 (19.9)	**< 0.001**
**Comments on the curriculum content**, n (%)				
Be attractive and in line with students’ learning objectives and requirements	342 (53.0)	190 (57.9)	152 (47.9)	**0.011**
Needs to be updated	103 (16.0)	51 (15.5)	52 (16.4)	0.767
Not very practical	145 (22.5)	68 (20.7)	77 (24.3)	0.279
Too difficult	73 (11.3)	30 (9.1)	43 (13.6)	0.077
Too easy	18 (2.8)	10 (3.0)	8 (2.5)	0.686
The continuity across course sections is inadequate	110 (17.1)	48 (14.6)	62 (19.6)	0.096
**Your expected evaluation mode for final exam**, n (%)				
Closed-book exam	34 (5.3)	19 (5.8)	15 (4.7)	0.058
Open-book exam	328 (50.9)	149 (45.4)	179 (56.5)	
Thesis or literature review	207 (32.1)	114 (34.8)	93 (29.3)	
Presentation using Microsoft PowerPoint	73 (11.3)	44 (13.4)	29 (9.1)	
Others	3 (0.5)	2 (0.6)	1 (0.3)	

*one student is excluded in this analysis because of not belonging to the 3 grades

In addition, seven Likert scale questions were designed to collect ordinal data regarding students’ opinions on the importance of the curriculum, the reasonability of the curricular provision, the extent of the conflict between the curriculum and your clinical work, scientific research, or other arrangements, the teaching effectiveness, the quantity and quality of teaching staff, and the teaching resource such as classroom equipment, as well as the overall management of postgraduate curriculum. Detailed descriptions of the Likert scale questions and their corresponding answers are listed in supplementary Table 1. The overall internal consistency reliability of the 7 Likert 5-point scales were acceptable, with the Cronbach’s alpha coefficient of 0.828. The Kaiser-Meyer-Olkin Measure of Sampling Adequacy was 0.862, Bartlett’s Test of Sphericity was significant (Approx. Chi-Square = 1680.864, p < 0.001), suggesting the sample size was adequate. In our present study, we focused on the students’ perceptions and behavioral intentions, so factor analysis was not performed.

### Statistics

The questionnaire data were analyzed using SPSS 23.0. Students were stratified by degree type (academic degree versus professional degree). The differences between groups were analyzed using Pearson chi-square test or Fisher’s exact test for categorical variables. The association between variables with ordinal values and degree type was analyzed using Mann-Whitney test. A heat map depicting student’s favorite and unfavorite courses of each degree type was generated using GraphPad Prism (version 8.0.2). The proportions of the last answers for the items “What is your opinion on the importance of the curriculum” (answer: not important at all) and “What is your assessment of the teaching effectiveness of our postgraduate curriculum” (answer: very bad) were too small. We therefore treated the last answer as the fourth answer for the two items in the distribution map.

## Results

Overall, 645 out of 908 full-time postgraduate students (71.0%) completed the questionnaire. As shown in Table 1, master-degree students accounted the majority of all included students (75.2%). Overall, academic-degree students and professional-degree students had different attitudes towards the curriculum provision, curricular content, and classroom organization.

### Attitude to curricular provision

With regard to “reasons for why curriculum matters”, professional-degree students were more likely to select the predefined answer “learning specialized knowledge and clinical skills” (p = 0.003), whereas academic-degree students were more inclined towards the answer “promoting multidisciplinary and interdisciplinary education and cultivating top talents” (p < 0.001). Subgroup analysis showed that the different preference for “learning specialized knowledge and clinical skills” was primarily observed among students in non-surgical departments (as shown in Table 2). Conversely, the variation in the inclination towards “promoting multidisciplinary and interdisciplinary education and cultivating top talents” was mainly observed among students in surgical departments.

**Table 2:** The attitudes of medical students to curriculum by degree type in surgical and non-surgical departments

Variable	Surgical departments (N = 149)			Non- Surgical departments (N = 245)		
	**Academic degree** (n = 52)	**Professional degree** (n = 97)	**P value**	**Academic degree** (n = 107)	**Professional degree** (n = 138)	**P value**
**Sex**, male, n (%)	36 (69.2)	59 (60.8)	0.309	28 (26.2)	34 (24.6)	0.785
Grade Level*						
2020, n (%)	47 (92.2)	80 (82.5)	0.139	83 (77.6)	103 (74.6)	0.711
2019, n (%)	1 (2.0)	11 (11.3)		13 (12.1)	16 (11.6)	
2018, n (%)	3 (5.9)	6 (6.2)		11 (10.3)	19 (13.8)	
**Postgraduate degree**, doctorate degree, n (%)	13 (25.0)	20 (20.6)	0.539	31 (29.0)	49 (35.5)	0.279
**Reasons why the curriculum is important**, n (%)						
Minimum credit requirements for applying for graduate degrees	30 (57.7)	51 (52.6)	0.550	64 (59.8)	77 (55.8)	0.528
Acquiring knowledge and skills for scientific research	44 (84.6)	79 (81.4)	0.627	87 (81.3)	114 (82.6)	0.793
Enhancing English proficiency for international academic exchange and academic paper writing	24 (46.2)	52 (53.6)	0.386	58 (54.2)	82 (59.4)	0.413
Acquiring specialized knowledge and clinical skills	24 (46.2)	42 (43.3)	0.738	34 (31.8)	70 (50.7)	**0.003**
Promoting multidisciplinary and interdisciplinary education and cultivating top talent	17 (32.7)	17 (17.5)	**0.035**	26 (24.3)	24 (17.4)	0.183
Developing knowledge of the humanities and social sciences	5 (9.6)	17 (17.5)	0.195	15 (14.0)	9 (6.5)	0.050
**Feedback on curriculum development**, n (%)						
Excessive compulsory courses and inadequate optional courses	11 (21.2)	40 (41.2)	**0.014**	20 (18.7)	41 (29.7)	**0.048**
Inadequate curriculum for developing scientific research abilities	31 (59.6)	46 (47.4)	0.156	66 (61.7)	73 (52.9)	0.169
Inadequate curriculum for acquiring clinical skills	22 (42.3)	42 (43.3)	0.907	36 (33.6)	51 (37.0)	0.591
Inadequate humanistic curriculum	11 (21.2)	23 (23.7)	0.723	20 (18.7)	21 (15.2)	0.470
Inadequate interdisciplinary curriculum	15 (28.8)	20 (20.6)	0.259	23 (21.5)	27 (19.6)	0.710
The course content has some overlap or repetition to some extent	15 (28.8)	15 (15.5)	0.052	35 (32.7)	28 (20.3)	**0.027**
**Comments on the curriculum content**, n (%)						
Be attractive and in line with students’ learning objectives and requirements	34 (65.4)	35 (36.1)	**0.001**	54 (50.5)	73 (52.9)	0.706
Needs to be updated	10 (19.2)	20 (20.6)	0.840	19 (17.8)	22 (15.9)	0.706
Not very practical	9 (17.3)	29 (29.9)	0.093	31 (29.0)	31 (22.5)	0.245
Too difficult	4 (7.7)	16 (16.5)	0.133	17 (15.9)	16 (11.6)	0.329
Too easy	2 (3.8)	2 (2.1)	0.611	3 (2.8)	4 (2.9)	1.000
The continuity across course sections is inadequate	6 (11.5)	24 (24.7)	0.055	15 (14.0)	23 (16.7)	0.570
**Your expected evaluation mode for final exam**, n (%)						
Closed-book exam	1 (1.9)	2 (2.1)	0.967	7 (6.5)	8 (5.8)	0.099
Open-book exam	33 (63.5)	57 (58.8)		49 (45.8)	73 (52.9)	
Thesis or literature review	14 (26.9)	30 (30.9)		34 (31.8)	49 (35.5)	
Presentation using Microsoft PowerPoint	4 (7.7)	8 (8.2)		16 (15.0)	8 (5.8)	
Others	0	0		1 (0.9)	0	

*1 student is excluded in this analysis because of not belonging to the 3 grades in subgroup of surgery departments

Regarding the question on “feedback on curriculum development”, it was found that among professional-degree students in both surgical departments and non-surgical departments, the answer “excessive compulsory courses and inadequate optional courses” was selected more frequently (p < 0.001). On the other hand, academic-degree students were more likely to choose the answers “inadequate in interdisciplinary curriculum” and “the course content has some overlap or repetition to some extent”.

**Fig. 1:**
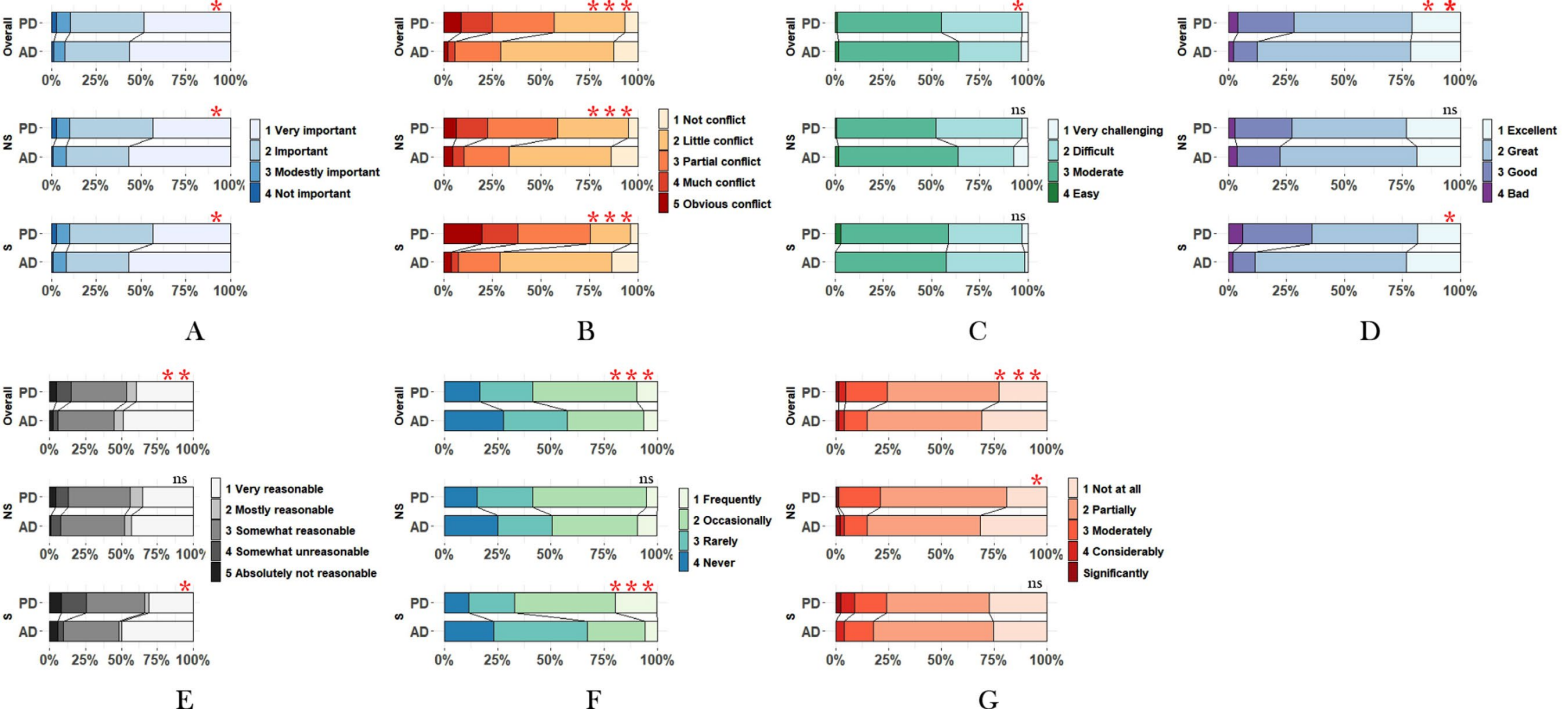
**A:** What is your opinion on the importance of the curriculum? **B:** Is there any conflict between the curriculum and your clinical work, scientific research, or other arrangements? **C:** What is the level of difficulty of the curriculum? **D:** What is your assessment of the teaching effectiveness of our postgraduate curriculum? **E:** What is your opinion on class attendance policy? **F:** How often do you arrive late or leave early in class? **G:** To what extent does using electronic devices in class affect your learning? Abbreviations: NS indicates non-surgical subgroup, and S indicates surgical subgroup. *p < 0.05, **p < 0.01, ***p < 0.001. ns means no significance

Overall, academic-degree students gave a higher rating for the importance of the curriculum, as depicted in Fig. 1A (p < 0.05 for overall group, non-surgical subgroup and surgical subgroup). Moreover, a greater proportion of professional-degree students thought the curriculum was conflict with clinical or research work (Fig. 1B. p < 0.001 for overall group, non-surgical subgroup and surgical subgroup).

### Attitude to curricular content

**Fig. 2:**
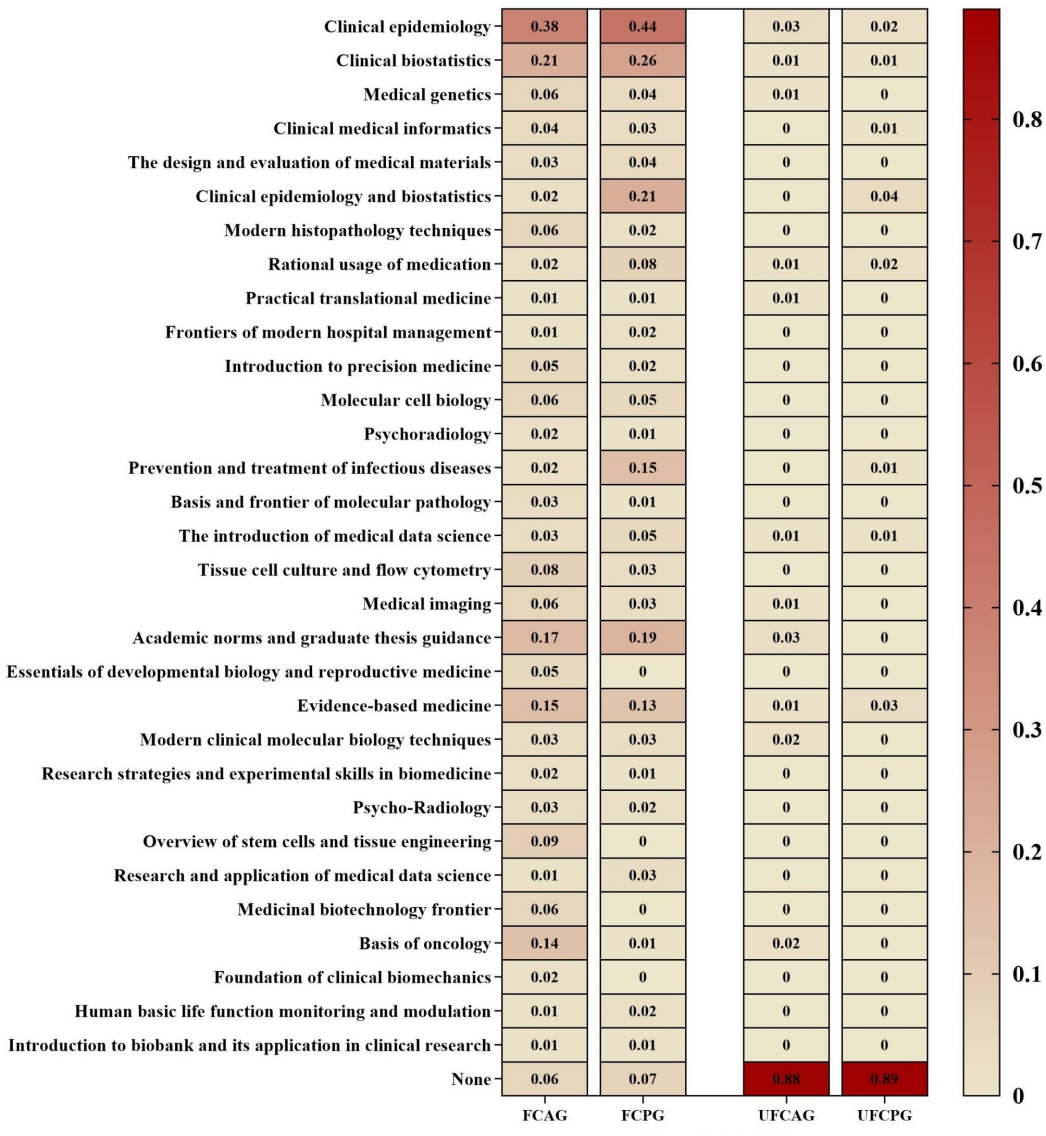
Heat map: FCAG: favorite courses in academic-degree group, FCPG: favorite courses in professional-degree group, UFCAG: unfavorite courses in academic-degree group, UFCPG: unfavorite courses in professional-degree group

For comments on the curricular content, a significantly higher proportion of academic-degree students (compared to professional-degree students) thought that the curriculum was attractive and in line with their learning objectives (p = 0.011), particularly in subgroup of surgical departments. Figure 2 displays a heat map indicating students’ favorite and unfavorite courses by degree type. Data showed that Clinical epidemiology and Clinical biostatistics ranked the top two in both academic-degree and professional-degree students. Additionally, postgraduate medical students showed a preference to Academic norms and postgraduate thesis guidance, as well as Evidence-based medicine. For the unfavorite courses, the majority of students in academic-degree and professional-degree subgroup (~ 90% in both subgroups) chose none.

Overall, there was a significant difference in the distribution of the level of curriculum difficulty between academic-degree and professional-degree students, with more professional-degree students rating the curriculum as difficult (Fig. 1C). As expected, professional-degree students rated the teaching effectiveness as less excellent(p < 0.01), particularly in the surgical subgroup(p < 0.05), as shown in Fig. 1D.

### Attitudes to the classroom organization

In terms of taking attendance, the data showed that more professional-degree students found it less reasonable (p < 0.01, Fig. 1E), and tended to arrive late or leave early from class (p < 0.001, Fig. 1F), especially in the surgical subgroup. It was striking that for these students who arrived late or leave early rarely, occasionally, or frequently, clinical work (55%, 355/501) and scientific research (16%, 103/501) ranked the top two among the reasons. Although there was not significant difference in the distribution of the frequency of using electronic devices during class, more professional-degree students thought that using electronic devices affected the learning effect, especially in non-surgical departments.

## Discussion

Most medical schools have performed evaluation systems to gather and analyze data on the specific curriculum [17, 18]. However, the attitudes of medical students towards the overall curriculum settings in academic-degree and professional-degree students is not unclear. In the present study, we analyzed the attitudes towards curriculum provision, content, and classroom organization by degree type, aiming to provide a comprehensive understanding of the students’ interest and to identify areas for potential improvement. These findings provide useful information for future medical education reforms in China.

One important finding was that professional-degree and academic-degree students had different priorities and expectations for the curriculum. Specifically, professional-degree students had a greater interest in the curriculum when it included specialized knowledge and clinical skills which was in line with the educational objectives of the current education program [3], while academic-degree students were more interested in the one which focused on multidisciplinary and interdisciplinary education and cultivating top talents. It’s accepted that multidisciplinary and interdisciplinary information is required for the research of disease mechanism [19, 20] and therefore comprehensive understanding of a certain disease [21, 22]. Therefore, it is important for both degrees to have multidisciplinary and interdisciplinary education. However, most of our participants were at master program and were in their first semester. Those students who pursing professional degree at the time of survey were in the process of transiting from undergraduate student to residents in our hospital, focusing on learning to manage medical paperwork and handle simple disease. When comparing this item among students with higher grades (grade 2019 and 2018), there were not significant differences in the proportion of “Promoting multidisciplinary and interdisciplinary education and cultivating top talent” between the two degree types. This phenomenon could be attributed to the fact that each departments arrange various teaching activities for postgraduate students, including lectures, teaching seminars, case analyses, and other methods to learn the latest advances in different disease types [23, 24]. As a result, for higher grade students who have received teaching activities across rotation departments, the difference in inadequate interdisciplinary curriculum on feedback on curriculum development was not significant (Supplementary Table 2). In fact, Since October 2022, multidisciplinary team discussions on a certain disease have been held monthly by hospital level, which is available to all students and doctors within this center, providing additional opportunities to learn through the process how to understand a specific type of disease and find an appropriate therapeutic schedule. Also, for higher grade students, the difference in “acquiring specialized knowledge and clinical skills” was not significant between professional and academic degree students (Supplementary Table 2). Therefore, opinions on the curriculum appear to change with the increase of academic year. In addition, professional-degree students expressed concerns that there were excessive compulsory courses and inadequate optional courses, while academic-degree students expressed that the course content has crossover or repetition to some extent. Therefore, it is important for curriculum makers and teaching administrators to take into account the diverse needs when revising the curriculum to ensure that it meets the needs of both types of students.

As expected, data showed that professional-degree students were less in favor of taking attendance policy in class when compared with academic-degree students. And professional-degree students reported that they arrive late or leave early from class more frequently, especially in the surgical subgroup. Wen et al. have reported that doctors in tertiary hospitals face heavy workload in China [25]. As a result, a greater proportion of professional-degree students in our present study felt that the curriculum was conflict with clinical work. When focused on non-surgical and surgical subgroups, the distribution of the frequency of “arrive late or leave early from class” was not different between academic-degree and professional-degree students in the non-surgical subgroup. This phenomenon might be attributed to the medical group policy at West China Hospital. The medical group comprises one or two senior physicians and around 5 residencies, which allows other residencies to manage patients if one residency (professional-degree student) in this group have curriculum to attend in non-surgical departments. However, in the surgical departments, surgery generally takes priority over the curriculum if there exists time conflict between surgery and curriculum, because the ongoing surgery could not stop. Therefore, the curriculum needs to be improved for professional-degree students, especially in surgery departments. Previous study has shown that male students have low attendance rate [26]. In our study, although male students were more common than female students in the surgical departments, the difference in the frequency of “arrive late or leave early” was not significant between male students and female students in both non-surgical and surgical subgroups. Taken together, our findings emphasize the importance of considering clinical rotation schedules while revising the curriculum for professional-degree students in surgical departments.

Another important finding was that there existed a significant correlation between taking attendance and teaching effectiveness (shown as the distribution diagram in Fig. 1, as determined by ordinal regression analysis, p < 0.001), with the lower frequency of “arrive late or leave early from class”, greater the teaching effectiveness. This finding was insightful for the curriculum reform, and was in line with previous studies [26, 27] that taking attendance was an important factor for passing exam. Also, previous study showed that students’ motivation and engagement significantly contribute to their learning success [28]. And mounted evidence have demonstrated that the online technology use such as websites and discussion boards [29], and animation and gamification [30] could increase students learning motivation and effectiveness. Meanwhile, asynchronous online learning was proved to be non-inferior to classroom-based teaching [31, 32]. Therefore, it might reasonable for professional students in the surgical departments to design more online classes such as self-learning modules as alternatives to cope with the time conflict between traditional classroom learning and clinical work, and check the self-learning effect using various evaluation method according to the online materials.

In fact, West China School of Medicine of Sichuan University has implemented a series of measures to address the issue of time conflicts between curriculum and clinical rotations for professional-degree students. For example, the majority of the courses are scheduled on evenings and weekends [6], and are arranged during the first academic year, in order to stagger clinical work. In addition, the teaching activities during department rotations are also arranged out of work-time. Taking Anesthesiology Department of West China Hospital as an example, teaching sessions are held on weekdays, mainly scheduled at either 07:15 a.m. or 19:00 p.m., aiming to minimize any disruption to regular clinical work [6]. Moreover, professional-degree students have not been assigned night duties during their first semester since 2019 in our school. In addition, some courses have been designed as Mini-class teaching, with the same content offered on different days of the week, thereby allowing students to choose the appropriate one according to their schedule. Therefore, another important measure to mitigate the conflict between curriculum and clinical work is to enhance the students’ subjective initiative in their learning.

### Strengths and limitations

Our study comprehensively analyzed the attitudes of postgraduate medical students toward the curriculum, with a large sample size and consideration of degree type. Also, our questionnaire was well-designed and contained detailed questions on the curriculum provision, content, and classroom organization. Our study provides valuable insights into the postgraduate students’ perceptions of their curricula, as well as evidence to support educational improvements in the curriculum to enhance teaching quality. Future study could investigate the specific reasons why more professional-degree students felt that the courses were conflict with routine work and arrived late or leaved early as compared with academic-degree students, despite a series of measures have been taken to address the issue of time conflicts between curriculum and clinical rotations for professional-degree students, as mentioned above. In addition, it would be valuable to investigate the actual factors influencing the attendance of professional-degree students. However, there are several limitations. Firstly, we did not distribute the questionnaire to all postgraduate medical students in West China School of Medicine of Sichuan University, as our focus was on new students (Grade 2020) who often take courses in their first academic year. Secondly, in the subgroup analysis, we focused exclusively on medical students who studied in the clinical departments. However, the way of grouping was reasonable, given that non-surgical and surgical departments confer both academic and professional degrees, and these two types of departments differ significantly in nature. Thirdly, this survey was conducted during the COVID-19 pandemic, potentially influencing the students’ attitude to the curriculum. This impact varied among different academic years. In the background that theoretical courses needed for applying for degree for postgraduate students are usually scheduled during the first academic year. Therefore, all the courses had been completed before COVID-19 pandemic for grade 2018. For grade 2019 and grade 2020, students experienced a blend of online and on-site courses because of the COVID-19 pandemic [33]. Finally, this study focused on postgraduate medical students from a single university in China, so future researches with larger sample size and including students from diverse universities and countries are needed to increase the generalizability of our findings. Therefore, our results are needed to be verified further.

## Conclusion

The attitudes towards the curriculum differ between professional-degree and academic-degree students, especially among students at the first academic year. It is important for curriculum makers and teaching administrators to take into account the diverse needs when revising the curriculum to ensure that it meets the needs of both types of students. This study provides benchmark data for the implementation of medical course reforms in China. In the future, various teaching methods such as outdoor learning and a combination of classroom learning could be considered as part of medical reforms, especially for professional-degree students.

### Electronic supplementary material

Below is the link to the electronic supplementary material.

Supplementary Material 1

Supplementary Material 2

## Data Availability

The datasets analyzed during the current study are within the study, and are available upon reasonable request from the corresponding author.
