# Supplementary material for: The attitudes of postgraduate medical students towards the curriculum by degree type: a large-scale questionnaire survey
Source: BMC Med Educ. 2023 Nov 16;23:869. doi: 10.1186/s12909-023-04846-5 (PMC10652528; doi:10.1186/s12909-023-04846-5)
Supplement: Supplementary file 1 — Supplementary Material 1 [file 12909_2023_4846_MOESM1_ESM.docx]

Supplementary table 1 The questions and their corresponding answers of the seven Likert scale

| **What is your opinion on the importance of the curriculum** | | | | |
| --- | --- | --- | --- | --- |
| 1-Very important | 2- Important | 3-Modestly important | 4- Not important | 5-Not important at all |
| **How reasonable are you with the curricular provision** | | | | |
| 1-Very reasonable | 2-Modestly reasonable | 3-Somewhat reasonable | 4-Somewhat unreasonable | 5-Absolutely not reasonable |
| **Is there any conflict between the curriculum and your clinical work, scientific research, or other arrangements** | | | | |
| 1-Not conflict | 2-Little conflict | 3-Partial conflict | 4-Much conflict | 5-Obvious conflict |
| **What is your assessment of the teaching effectiveness of our postgraduate curriculum** | | | | |
| 1-Excellent | 2-Great | 3-Good | 4-Bad | 5-Very bad |
| **How satisfied or dissatisfied are you with the quantity and quality of teaching staff** | | | | |
| 1-Very satisfied | 2-Satisfied | 3-Unsure | 4-Dissatisfied | 5-Very dissatisfied |
| **How satisfied or dissatisfied are you with the teaching resource such as classroom equipment** | | | | |
| 1-Very satisfied | 2-Satisfied | 3-Unsure | 4-Dissatisfied | 5-Very dissatisfied |
| **How satisfied or dissatisfied are you with the overall management of postgraduate curriculum** | | | | |
| 1-Very satisfied | 2-Satisfied | 3-Unsure | 4-Dissatisfied | 5-Very dissatisfied |
