# Supplementary material for: The attitudes of postgraduate medical students towards the curriculum by degree type: a large-scale questionnaire survey
Source: BMC Med Educ. 2023 Nov 16;23:869. doi: 10.1186/s12909-023-04846-5 (PMC10652528; doi:10.1186/s12909-023-04846-5)
Supplement: Supplementary file 2 — Supplementary Material 2 [file 12909_2023_4846_MOESM2_ESM.docx]

**Supplemental table 2 The Attitudes of medical students to curriculum by degree type in low grade and high grade**

| **Variable** | **Low grade (grade 2020) (N=534)** | | | **High grade (grades 2018 and 2019) (N=110)** | | |
| --- | --- | --- | --- | --- | --- | --- |
|  | **Academic degree** (n=276) | **Professional degree** (n=258) | **P value** | **Academic degree** (n=51) | **Professional degree** (n=59) | **P value** |
| **Sex**, male，n (%) | 88 (31.9) | 78(30.2) | 0.680 | 10 (19.6) | 26(44.1) | 0.006 |
| **Postgraduate degree,** master degree, n (%) | 220(79.7) | 207(80.2) | 0.880 | 29 (56.9) | 28 (47.5) | 0.325 |
| **Reasons why the curriculum is important**, n (%) | | | | | | |
| Minimum credit requirements for applying for graduate degrees | 151 (54.7) | 127 (49.2) | 0.205 | 33 (64.7) | 42 (71.2) | 0.467 |
| Acquiring knowledge and skills for scientific research | 232 (84.1) | 216 (83.7) | 0.916 | 43 (84.3) | 48 (81.4) | 0.682 |
| Enhancing English proficiency for international academic exchange and academic paper writing | 143 (51.8) | 151 (58.5) | 0.119 | 29 (56.9) | 33 (55.9) | 0.922 |
| Acquiring specialized knowledge and clinical skills | 105 (38.0) | 133 (51.6) | **0.002** | 17 (33.3) | 22 (37.3) | 0.665 |
| Promoting multidisciplinary and interdisciplinary education and cultivating top talent | 83 (30.1) | 46 (17.8) | **0.001** | 15 (29.4) | 9 (15.3) | 0.073 |
| Developing knowledge of the humanities and social sciences | 43 (15.6) | 25 (9.7) | **0.041** | 3 (5.9) | 9 (15.3) | 0.116 |
| **Feedback on curriculum development**, n (%) | | | | | | |
| Excessive compulsory courses and inadequate optional courses | 46 (16.7) | 95 (36.8) | **<0.001** | 9(17.6) | 15 (25.4) | 0.325 |
| Inadequate curriculum for developing scientific research abilities | 142 (51.4) | 132 (51.2) | 0.947 | 25 (49.0) | 36 (61.0) | 0.207 |
| Inadequate curriculum for acquiring clinical skills | 89 (32.2) | 100 (38.8) | 0.116 | 16 (31.4) | 22 (37.3) | 0.515 |
| Inadequate humanistic curriculum | 61 (22.1) | 42 (16.3) | 0.088 | 12 (23.5) | 10 (16.9) | 0.390 |
| Inadequate interdisciplinary curriculum | 79 (28.6) | 56 (21.7) | 0.066 | 20 (39.2) | 15 (25.4) | 0.121 |
| The course content has some overlap or repetition to some extent | 100 (36.2) | 55 (21.3) | **<0.001** | 8 (15.7) | 8 (13.6) | 0.752 |
| **Comments on the curriculum content**, n (%) | | | | | | |
| Be attractive and in line with students’ learning objectives and requirements | 161 (58.3) | 120 (46.5) | **0.006** | 28 (54.9) | 32 (54.2) | 0.944 |
| Needs to be updated | 40 (14.5) | 42 (16.3) | 0.567 | 11 (21.6) | 10 (16.9) | 0.539 |
| Not very practical | 56(20.3) | 62 (24.0) | 0.298 | 12 (23.5) | 15 (25.4) | 0.818 |
| Too difficult | 26 (9.4) | 37 (14.3) | 0.078 | 4 (7.8) | 6(10.2) | 0.749 |
| Too easy | 9 (3.3) | 4 (1.6) | 0.200 | 1 (2.0) | 4 (6.8) | 0.370 |
| The continuity across course sections is inadequate | 42 (15.2) | 57 (22.1) | **0.041** | 6 (11.8) | 5 (8.5) | 0.566 |
